# What is the impact of *PCSK9* rs505151 and rs11591147 polymorphisms on serum lipids level and cardiovascular risk: a meta-analysis

**DOI:** 10.1186/s12944-017-0506-6

**Published:** 2017-06-12

**Authors:** Chengfeng Qiu, Pingyu Zeng, Xiaohui Li, Zhen Zhang, Bingjie Pan, Zhou Y. F. Peng, Yapei Li, Yeshuo Ma, Yiping Leng, Ruifang Chen

**Affiliations:** 1 Xiangya school of Pharmaceutical Sciences, Central South University, Changsha, China; 2 Center for Vascular Disease and Translational Medicine, The Third Xiangya Hospital of Central South University, Changsha, China; 3 Department of Cardiology, The Third Xiangya Hospital of Central South University, Changsha, China

**Keywords:** Proprotein convertase subtilisin/kexin type 9, Polymorphisms, Cardiovascular risk, Lipids, Meta-analysis

## Abstract

**Background:**

*PCSK9* rs505151 and rs11591147 polymorphisms are identified as gain- and loss-of-function mutations, respectively. The effects of these polymorphisms on serum lipid levels and cardiovascular risk remain to be elucidated.

**Methods:**

In this meta-analysis, we explored the association of *PCSK9* rs505151 and rs11591147 polymorphisms with serum lipid levels and cardiovascular risk by calculating the standardized mean difference (SMD) and odds ratios (OR) with 95% confidence intervals (CI).

**Results:**

Pooled results analyzed under a dominant genetic model indicated that the *PCSK9* rs505151 G allele was related to higher levels of triglycerides (SMD: 0.14, 95% CI: 0.02 to 0.26, *P* = 0.021, I^2^ = 0) and low-density lipoproteins cholesterol (LDL-C) (SMD: 0.17, 95% CI: 0.00 to 0.35, *P* = 0.046, I^2^ = 75.9%) and increased cardiovascular risk (OR: 1.50, 95% CI: 1.19 to 1.89, *P* = 0.0006, I^2^ = 48%). The rs11591147 T allele was significantly associated with lower levels of total cholesterol (TC) and LDL-C (TC, SMD: -0.45, 95% CI: -0.57 to −0.32, *P* = 0.000, I^2^ = 0; LDL-C, SMD: -0.44, 95% CI: -0.55 to −0.33, *P* = 0.000, I^2^ = 0) and decreased cardiovascular risk (OR: 0.77, 95% CI: 0.60 to 0.98, *P* = 0.031, I^2^ = 59.9) in Caucasians.

**Conclusions:**

This study indicates that the variant G allele of *PCSK9* rs505151 confers increased triglyceride (TG) and LDL-C levels, as well as increased cardiovascular risk. Conversely, the variant T allele of rs11591147 protects carriers from cardiovascular disease susceptibility and lower TC and LDL-C levels in Caucasians. These findings provide useful information for researchers interested in the fields of *PCSK9* genetics and cardiovascular risk prediction not only for designing future studies, but also for clinical and public health applications.

## Background

Cardiovascular disease (CVD) is the leading cause of death and contributes substantially to the heavy disease burden worldwide [1]. It is a complex and multifactorial disease caused by the interaction of vascular risk factors, as well as environmental and genetic factors. An elevated level of serum low-density lipoprotein cholesterol (LDL-C), which is the most common and clinically relevant dyslipidemia, has been well established as a major risk factor for cardiovascular disease [2]. Genetic susceptibility to cardiovascular disease and dyslipidemia has been researched extensively and there is mounting evidence demonstrating that genetic variants are associated with CVD and dyslipidemia.

Proprotein convertase subtilisin/kexin type 9 (PCSK9), which was identified as the ninth member of the proprotein convertase family in 2003, plays a key role in lipid metabolism, and has emerged as an important modulator of cardiovascular health [3]. Insights into the physiological function of PCSK9 were derived initially from the recognition of functional mutations in the *PCSK9* gene that cause an autosomal dominant form of hypercholesterolemia (ADH). Extensive research into the function of PCSK9 has since been conducted. To date, the best characterized property of PCSK9 is its ability to enhance the intracellular degradation of the LDL receptor (LDLR), which mediates approximately 70% of LDL-C clearance. Secreted PCSK9 in the circulation effectively binds to the LDLR on the surface of hepatocytes, thereby targeting the LDLR for lysosomal degradation and preventing recycling to the hepatocyte surface, thus leading to considerable elevation in LDL-C levels [4].

The *PCSK9* gene is located on the small arm of chromosome 1p32.3 and comprises 12 exons and 11 introns [5]. It is highly polymorphic, with a total of 163 mutations identified so far. These mutations and polymorphisms are distributed in all PCSK9 domains. Although the PCSK9 gene has been found to cause only 2% of ADH; its numerous nonsynonymous variants are functionally relevant in cholesterol regulation and result in considerable changes in blood cholesterol levels in the general population much more than do LDLR or APOB polymorphisms, which are the other two common genes that cause ADH [6]. These functional variants are classified as two categories: gain-of-function (GOF) mutations associated with hypercholesterolemia phenotype and loss-of-function (LOF) mutations, which cause hypocholesterolemia [7]. PCSK9 rs505151 (−23968A > G, E670G) is a common GOF-mutation, this SNP is located in exon 12, and results in an amino acid substitution from glutamate to glycine at position 670 [8]. The *PCSK9* rs11591147 (137G > T, R46L) variant contains a replacement mutation (arginine to leucine at position 46) located in exon 1. This relatively rare variant is considered to be a LOF-mutation of PCSK9 [9]. Numerous studies in different ethnic group have been performed to investigate the impact of the rs505151 and rs11591147 variants on plasma lipid homeostasis and associations with the incidence of cardiovascular risk; however, the findings to date are inconsistent. Variants are unequally distributed in different ethnic group and their impact vary in different populations. Therefore, we conducted the current meta-analysis of all eligible studies to provide robust evidence of the associations of rs505151 and rs11591147 variation with lipid traits and susceptibility to CVD.

## Methods

### Search strategy, study selection and data extraction

The current meta-analysis was performed according to the principles proposed by the Human Genome Epidemiology Network (HuGeNet) HuGE Review Handbook of Genetic Association Studies [10, 11].

Studies dealing with the associations of the two SNPs (rs505151 and rs11591147) with plasma lipids levels and risk of cardiovascular disease in humans were considered eligible. Relevant studies were searched in PubMed, Chinese National Knowledge Infrastructure and WANFANG database. The search work was last updated on September 1, 2016. The following three groups of keywords we performed by searching MEDLINE (via the PubMed gateway): “proprotein convertase subtilisin/kexin type 9” OR PCSK9 OR “neural apoptosis-regulated convertase 1” OR NARC1, polymorphism OR SNP OR “single nucleotide polymorphism” OR variant OR variation OR mutation, lipid OR dyslipidemia OR “coronary heart disease” OR “myocardial infarction” OR “coronary artery disease” OR “ischemic heart disease” OR “acute coronary syndrome” OR “CAD” OR “CHD”. References from the retrieved articles and previous meta-analysis were searched manually for additional qualified studies.

The studies eligible for the meta-analysis must meet all the following inclusion criteria: (i) case-control or cohort studies; (ii) contained rs505151 and/or rs11591147 genotype data; (iii) adequate data for calculating the standardized mean difference (SMD) and odds ratios (ORs) and correspond 95% confidence intervals (CIs). Exclusion criteria were as follows: (i) studies did not provide sufficient data to extract the information we needed; (ii) case report, review, meta-analysis, cell line and animal experiment studies; (iii) repeated publication about the same population.

Two investigators (Qiu and Li) screened all the records and extracted data independently, the third investigator (Zhang) was involved in discussing to avoid bias when there were disagreements between Qiu and Li. Following information was extracted from each of the eligible studies: first author, year of publication, ethnic groups of the patients, type of study, sample size, genotyping method, age, sex, minor allelic frequency (MAF); Hardy-Weinberg equilibrium (HWE).

### Statistical analysis

The deviations from the HWE for the *PCSK9* rs505151 and rs11591147 genotype distributions were assessed by Fisher’s exact test. A *p* < 0.05 for the test was considered deviated from the HWE. The pooled SMD with 95% confidence interval (CI) was applied to calculate the differences of plasma lipid levels between different genotypes. The OR and corresponding 95% CI were used to evaluate the strength of the association between the polymorphisms of two SNPs and cardiovascular risk. Dominant genetic model was conducted to assess the genetic associations, the reasons for the choice are as follows: (i) *PCSK9* rs505151 and rs11591147 polymorphisms are rare in human and low MAF were presented in candidate gene studies, on the premise that the difference between carrying one and two copies of the genetic variant is likely to have less effect on the effect size (OR and SMD), perhaps a dominate mode is most reasonable. (ii) For some studies, only dominant genetic data were available; (iii) one model does not require adjustment for multiple hypotheses (which is necessary when different models are used); however, dominant model is commonly used in genetic association synopses.

Between-study heterogeneity was assessed by the chi-square-based Q test and I^2^ statistics [12]. A *p* < 0.10 for the Q test was considered statistically significant. For I^2^, which describes the proportion variation in point estimates that is due to variance rather than within-study error, the value of I^2^ ranged from 0 to100% indicates different degree of heterogeneity (0 to 25%: no heterogeneity; 25 to 50%: moderate heterogeneity; 50 to 75%: large heterogeneity; and 75 to 100%: extreme heterogeneity). Meta-regression, meta-sensitivity and subgroup analysis were conducted to explore the sources of heterogeneity when *p* < 0.10 for the Q test. SMD, ORs and corresponding 95%CI were calculated by performing fixed effect meta-analysis when the heterogeneity was under the moderate degree or not exist; in otherwise, the analysis model reduced to a random effect meta-analysis. The choice of this model was suggested mainly by the heterogeneity mostly expected in genetic association studies. Meanwhile, the potential bias was assessed by statistical evaluation with Begg’s rank correlation [13] and Egger’s linear regression tests [14] while the numbers of single studies reached three or more. For each variant, a meta-analysis was performed if at least two independent studies were available.

The α level of significance was set at 0.05, except for the Q-test (0.10).

All statistical analyses were performed with with STATA/SE.12.0 (StataCorp, College station, Texas, USA), Review Manager Version 5.3 (The Nordic Cochrane Center, Copenhagen, Denmark).

### Assessment of cumulative evidence

The Venice criteria was applied to assess the credibility of each nominally statistically significant association identified by meta-analysis [15]. Three categories were defined according to the amount of evidence, extent of replication and protection from bias, and also generates a composite assessment of ‘strong’, ‘moderate’ or ‘weak’ epidemiological credibility.

1. Amount of evidence, mainly based on the study sample size, was graded by the sum of subjects carrying the variant allele (total number of cases and controls), category “A” corresponds to a sample size over 1000, “B” and “C” correspond to 100–1000 and <100, respectively.
2. Replication of genetic associations were depended upon the between-study inconsistency defined by I^2^, where I^2^ < 25% was considered as “A”, 25–50% and >50% were identified as “B” and “C”, respectively.
3. Protection from bias was graded as “A” if there was no notable bias or may not affect the presence of the association; in category “B”, bias could be present or there was considerable missing information on the generation of evidence; in category “C”, demonstrable bias that can affect the presence or absence of the association.

Followed the three letters stated above, evidence was categorized as “strong” (A grades only), “weak” (one or more C grades) or “moderate” (all other combinations).

The quality of summary evidence for no association was also assessed in the meta-analysis. We calculated the power instead of the sample size; the other aspects including replication and protection from bias were also accounted for according to the Venice criteria [15]. The power was identified as “A” if the power were ≥90%, “B” and “C” correspond to 80–90% and <80%, respectively [16]. Evidence was categorized as “strong” (A grade only), “weak” (one or more C grades) or “moderate” (all other combinations).

## Results

### Characteristics of eligible studies

**Fig. 1:**
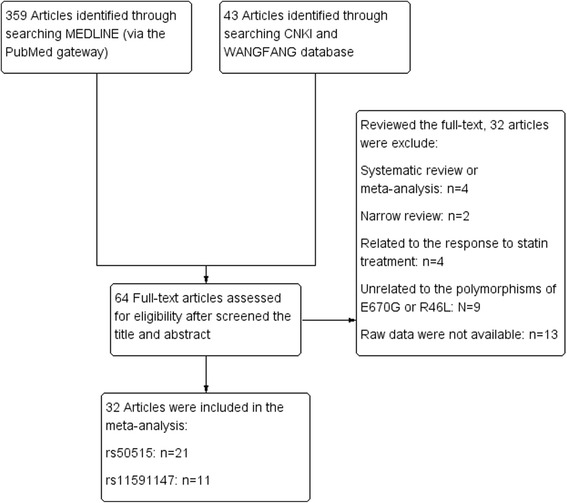
Flow diagram of the study selection process

A total of 32 studies met the inclusion criteria and were included in the final meta-analysis, the process of study selection were shown in Fig. 1. The number of studies which were included in meta-analysis ranged from 2 to 20. With regard to P*CKS9* rs505151 polymorphism, 15 articles comprising 14,451 subjects were identified from the initial search corresponded to the plasma lipid levels, 3373 (23%) were Asians and 11,078 (77%) were Caucasians. The MAF ranged greatly from 2.1%to 14.7%. Genotype distributions in four studies deviated from HWE and two studies did not provide sufficient data to evaluate genotype distributions. There were 12 case-control articles including 11,203 subjects related to the association between rs505151 and cardiovascular disease risk. The constituent ratios of Asians and Caucasians were 23% and 77% respectively. In this group, the MAF varied from 3.8% to 7.5%, and it lower than the reported frequency (10%). Among them, only one study of the genotypes distribution in controls deviated from HWE, and one study was failed to assess the genotype distributions. We identified 8 eligible articles encompassing 17,090 subjects to study the association between *PCSK9* rs11591147 variation and plasma lipid levels, most cases were Caucasians (98%), MAF of this group higher than the reported frequency (0.6%) and it varied from 1.6% to 25.3%. Except four articles that could not to be assessed the distribution of genotypes, it did not deviated from HWE in the rest 6 articles. Four case-control studies and three cohort studies revealed the association between *PCSK9* rs11591147 polymorphism and cardiovascular disease risk, a total of 60,677 subjects included in this group and all of them were Caucasians. The MAF ranged from 1% to 1.7%. The distribution of genotypes in controls of these studies did not deviated from HWE, except one study that could not to be evaluated. The details of characteristics of each individual study were demonstrated in Table 1 and Table 2.

**Table 1:** Characteristics of studies related to the associations of rs50515 and rs11591147 with lipids levels

Author	Year	Ethnicity	Type of study	Sample size	SNP	Genotyping method	Age(year)	Sex(M/F)	MAF (%)	HWE
He, X M [27]	2016	Asians	C-C	233	rs505151	PCR-RFLP	63.95 ± 12.35	130/103	13.9	0.015
Jeenduang, N1 [28]	2015	Asians	Cohort	307	rs505151	PCR-RFLP	NA	97/210	1.95	0.727
Jeenduang, N2 [28]	2015	Asians	Cohort	188	rs505151	PCR-RFLP	NA	38/150	1.06	0.883
Yuqian, Mo [29]	2015	Asians	C-C	100	rs505151	TaqMan	56.4 ± 11.7	58/42	7.19	0.000
Anderson, J M1 [30]	2014	Caucasians	C-C	171	rs505151	PCR-RFLP	47 ± 7	108/55	12.6	NA
Anderson, J M2 [30]	2014	Caucasians	C-C	163	rs505151	PCR-RFLP	55 ± 10	127/44	14.7	NA
Slimani, A [31]	2014	Caucasians	C-C	258	rs505151	PCR-RFLP	Case:55 (52–65) Control:61 (55–67)	212/46	0.88	0.000
Mayne, J [32]	2013	Caucasians	Cohort	207	rs505151	PCR-RFLP	NA	NA	0.036	0.591
Aung, L H1 [33]	2013	Asians	Cohort	567	rs505151	PCR-RFLP	45.13 ± 13.35	456/111	0.021	0.60
Aung, L H2 [33]	2013	Asians	Cohort	785	rs505151	PCR-RFLP	47.36 ± 14.34	573/212	0.026	0.453
Meng yanhui [34]	2011	Asians	Cohort	165	rs505151	PCR-RFLP	66.49 ± 9.92	102/63	0.058	0.430
Zeng jian [35]	2011	Asians	C-C	212	rs505151	PCR-RFLP	NA	NA	0.123	0.020
Norata, G D [36]	2010	Caucasians	Cohort	1541	rs505151	Taqman	54.71 ± 10.97	621/923	0.024	0.32
Hsu, L A1 [37]	2009	Asians	C-C	202	rs505151	PCR-RFLP	55.6 ± 10.5	171/31	0.046	0.513
Hsu, L A2 [37]	2009	Asians	C-C	614	rs505151	PCR-RFLP	45.9 ± 10.4	325/289	0.060	0.381
Polisecki, E [38]	2008	Caucasians	Cohort	5416	rs505151	Taqman	75.64 ± 3.38	2620/2795	0.030	0.072
Scartezini, M [39]	2007	Caucasians	Cohort	2444	rs505151	PCR-RFLP	50–61	2444(M)	0.065	0.788
Evans, D [40]	2006	Caucasians	Cohort	506	rs505151	PCR-RFLP	44 ± 14	239/267	0.050	0.111
Chen, SN [41]	2005	Caucasians	Cohort	372	rs505151	PCR-RFLP	58.8 ± 7.7	310/62	0.074	0.000
Jeenduang, N1 [28]	2015	Asians	Cohort	97	rs11591147	PCR-RFLP	NA	97(M)	0.031	0.753
Jeenduang, N2 [28]	2015	Asians	Cohort	209	rs11591147	PCR-RFLP	NA	NA	0.019	0.778
Bonnefond, A [42]	2015	Caucasians	C-C	4319	rs11591147	Metabochip	46.7 ± 10.0	2272/2246	0.020	0.179
Saavedra, YG [43]	2014	Caucasians	Cohort	560	rs11591147	PCR-RFLP	37.0 ± 13.9	329/231	0.016	0.699
Guella, I [44]	2010	Caucasians	c-c	3453	rs11591147	Taqman	39.6 ± 4.9	3039/414	NA	NA
Strom, T B [45]	2010	Caucasians	Cohort	1130	rs11591147	PCR-RFLP	34 ± 13.3	536/594	NA	NA
Huang, C C [46]	2009	Caucasians	Cohort	1828	rs11591147	Taqman	25.5 ± 3.4	852/916	NA	NA
Humphries, S E [47]	2009	Caucasians	Cohort	81	rs11591147	PCR-RFLP	56 ± 3.6	81(M)	0.253	0.840
Polisecki, E [38]	2008	Caucasians	Cohort	5413	rs11591147	Taqman	75.64 ± 3.38	2619/2794	0.018	0.069

*Abbreviations: C-C* case-control, *M* male, *F* female, *MAF* minor allelic frequencies, *HWE* Hardy-Weinberg equilibrium, *NA* not assess

**Table 2:** Characteristics of the studies related to the associations of rs50515 and rs11591147 with cardiovascular risk

Author	Year	Ethnicity	Subgroup	Type of study	Sample size	SNP	Genotyping method	Age(year)	Sex (M/F)	MAF (%)	HWE (p)
He, X M [27]	2016	Asians	CHD	C-C	472	rs505151	PCR-RFLP	62.80 ± 12.76	251/221	0.056	0.07
Yuqian, Mo [29]	2015	Asians	CHD	C-C	200	rs505151	Taqman	55.55 ± 10.98	114/86	0.040	0.677
Yangchun [48]	2015	Asians	MI	C-C	342	rs505151	PCR-RELP	58.38 ± 6.45	200/144	0.038	0.589
Slimani, A1 [31]	2014	Asians	CHD	C-C	258	rs505151	PCR-RFLP	Case:55 (52–65) Control:61 (55–67)	212/46	0.068	0.552
Slimani, A3 [31]	2014	Caucasians	IS	C-C	346	rs505151	PCR-RELP	Case:66 (54.5–76.5) Control:49 (45–55)	237/109	0.073	0.812
Lei, Jing [49]	2014	Caucasians	IS	C-C	756	rs505151	SNaPshot	61.91 ± 112	425/331	0.056	0.925
Meng yanhui [34]	2011	Asians	CHD	C-C	345	rs505151	PCR-RFLP	65.37 ± 13.06	200/180	0.039	0.587
Zeng jian [35]	2011	Asians	CHD	C-C	396	rs505151	PCR-RFLP	NA	NA	0.057	0.055
Guella, I [44]	2010	Caucasians	MI	C-C	4643	rs505151	PCR-RELP	36.69 ± 4.92	3317/420	NA	NA
Hsu, L A [37]	2009	Asians	CHD	C-C	816	rs505151	PCR-RFLP	48.30 ± 11.23	496/320	0.060	0.381
Scartezini, M [39]	2007	Caucasians	CHD	C-C	2065	rs505151	PCR-RFLP	56.16 ± 3.41	2065(M)	0.033	0.465
Abboud, S [50]	2007	Caucasians	IS	C-C	564	rs505151	TaqMan	Case(53.5) Control(70.3)	NA	0.075	0.012
Saavedra, Y G3 [43]	2014	Caucasians	Cohort	Cohort	560	rs11591147	PCR-RFLP	37.0 ± 13.9	329/231	0.016	0.699
Benn, M1 [21]	2010	Caucasians	C-C	C-C	10,032	rs11591147	Taqman	58.0 (44–690	4514/5518	0.012	0.676
Benn, M2 [21]	2010	Caucasians	C-C	C-C	26,013	rs11591147	Taqman	59.8 (51–69)	12746/13267	0.014	0.334
Benn, M3 [21]	2010	Caucasians	C-C	C-C	9654	rs11591147	Taqman	60.0 (51–69)	5599/4055	0.012(total)	0.748
Guella, I [44]	2010	Caucasians	C-C	C-C	4733	rs11591147	Taqman	39.6 ± 4.9	3039/414	NA	NA
Huang, C C [46]	2009	Caucasians	Cohort	Cohort	1828	rs11591147	Taqman	25.52 ± 3.41	882/946	0.170	0.505
Polisecki, E1 [38]	2008	Caucasians	Cohort	Cohort	5413	rs11591147	Taqman	75.64 ± 3.38	2619/2794	0.018	0.840
Scartezini, M [39]	2007	Caucasians	C-C	C-C	2444	rs11591147	PCR	50–61	2444(M)	0.010	0.074

*Abbreviations: CHD* coronary heart disease, *MI* myocardial infarction, *IS* ischemic stroke, *C-C* case-control, *M* male, *F* female, *MAF* minor allelic frequencies, *HWE* Hardy-Weinberg equilibrium, *NA* not assess

**Table 3:** Associations of rs505151 and rs11591147 variants with serum lipids levels and blood pressure

SNP	variant allele	Lipid traits or BP	Sub- group	No. of Study	Sample size	effect model	SMD	95% CI	P	I2%	Phet	Begg ‘(P)	Egger’s (P)	Venice criteria	Level of evidence
rs505151	G	TC	Asians	11	3318	random	0.16	(−0.06,0.38)	0.151	62.9	0.003	0.815	0.407	BCA	weak
rs505151	G	TC	Caucasians	9	9664	random	0.03	(−0.14,0.21)	0.716	76.0	0.000	0.532	0.151	BCA	weak
rs505151	G	TC	Overall	20	12,982	random	0.09	(−0.04,0.22)	0.190	68.5	0.000	0.846	0.601	ACA	weak
rs505151	G	TG	Asians	12	3373	fixed	0.14	(0.02,0.26)	0.021	0.0	0.529	0.586	0.411	BAA	Moderate
rs505151	G	TG	Caucasians	7	3183	fixed	−0.09	(−0.21,0.03)	0.127	23.9	0.247	0.099	0.294	BAA	Moderate
rs505151	G	TG	Overall	19	6556	fixed	0.02	(−0.06,0.11)	0.596	30.0	0.112	0.472	0.623	BBA	Moderate
rs505151	G	LDL-C	Asians	11	3335	random	0.28	(−0.07,0.62)	0.113	84.2	0.000	0.312	0.190	BCA	weak
rs505151	G	LDL-C	Caucasians	8	4248	random	0.11	(−0.02,0.24)	0.111	37.6	0.129	0.805	0.710	BBA	Moderate
rs505151	G	LDL-C	Overall	19	7583	random	0.17	(0.00,0.35)	0.046	75.9	0.000	0.151	0.092	BCA	weak
rs505151	G	HDL-C	Asians	11	3273	fixed	−0.16	(−0.19,0.06)	0.340	44.1	0.065	0.245	0.560	BBA	Moderate
rs505151	G	HDL-C	Caucasians	8	4248	fixed	0.02	(−0.08,0.12)	0.673	0.0	0.803	0.095	0.091	BAA	Moderate
rs505151	G	HDL-C	Overall	19	7521	fixed	−0.01	(−0.09,0.07)	0.792	18.7	0.230	0.103	0.154	BAA	Moderate
rs505151	G	DBP	Mixed	8	5079	fixed	−0.08	(−0.21,0.05)	0.224	0.0	0.692	0.805	0.810	BAA	Moderate
rs505151	G	SBP	Mixed	8	5079	fixed	−0.06	(−0.19,0.06)	0.332	7.0	0.376	0.805	0.643	BAA	Moderate
rs11591147	T	TC	Asians	2	306	random	−0.50	(−1.44,0.44)	0.299	66.5	0.084			CCA	weak
rs11591147	T	TC	Caucasians	6	9496	random	−0.45	(−0.57,-0.32)	0.000	0.0	0.936	0.851	0.913	BAA	Moderate
rs11591147	T	TC	Overall	8	9802	random	−0.45	(−0.57,-0.33)	0.000	0.0	0.733	1.000	0.869	BAA	Moderate
rs11591147	T	TG	Asians	2	306	fixed	−0.43	(−0.97,0.11)	0.119	0.0	0.447			CAA	weak
rs11591147	T	TG	Caucasians	5	9254	fixed	0.00	(−0.11,0.11)	0.996	0.0	0.685	0.624	0.436	BAA	Moderate
rs11591147	T	TG	Overall	7	9560	fixed	−0.02	(−0.13,0.09)	0.747	0.0	0.522	0.176	0.102	BAA	Moderate
rs11591147	T	LDL-C	Asians	2	306	fixed	−0.53	(−1.07,0.01)	0.053	0.0	0.436				
rs11591147	T	LDL-C	Caucasians	5	10,299	fixed	−0.44	(−0.55,-0.33)	0.000	0.0	0.548	0.142	0.125	BAA	Moderate
rs11591147	T	LDL-C	Overall	7	10,605	fixed	−0.44	(−0.55,-0.33)	0.000	0.00.0	0.707	0.293	0.206	BAA	Moderate
rs11591147	T	HDL-C	Mixed	7	9560	fixed	0.10	(−0.01,0.21)	0.074	35.4	0.158	0.453	0.026	BBB	Moderate
rs11591147	T	DBP	Mixed	5	2797	random	3.60	(−0.71,7.9)	0.101	77.4	0.001	0.624	0.318	CCA	weak
rs11591147	T	SBP	Mixed	5	2797	random	1.79	(−2.78,6.37)	0.442	45.6	0.118	0.624	0.306	CBA	weak

*Abbreviations: SMD* standardized mean difference, *95%C* 95% confidence interval, *TC* cholesterol, *TG* triglyceride, *LDL-C* low-density lipoprotein cholesterol, *HDL-C* high density lipoprotein cholesterol, *SBP* systolic blood pressure, *DBP* diastolic blood pressure

**Table 4:** Associations of rs505151 and rs11591147 variants with cardiovascular risk

SNP	Variant allele	Subgroup	No. of Study	Sample size	effect model	OR	95% CI	*P* value	I^2^%	P_het_	Begg’ (P)	Egger’s (P)	Venice criteria	Level of evidence
rs505151	G	Asians	8	1672	random	1.59	(1.12,2.27)	0.009	60	0.01	0.805	0.437	BCA	Weak
rs505151	G	Caucasians	4	2369	random	1.31	(1.04,1.65)	0.02	0	0.45	0.042	0.229	BAA	Moderate
rs505151	G	Overall		4041	random	1.50	(1.19,1.89)	0.0006	48	0.03	0.493	0.144	BBA	Moderate
rs11591147	T	Caucasians	9	66,090	random	0.77	(0.60,0.98)	0.031	59.9	0.015	0.216	0.165	BCA	Weak

*Abbreviations: OR* odds ratio, *95% CI* 95% confidence interval

### Quantitative synthesis of data

#### Associations of *PCSK9* rs505151 and rs11591147 polymorphisms with plasma lipid levels and blood pressure

**Fig. 2:**
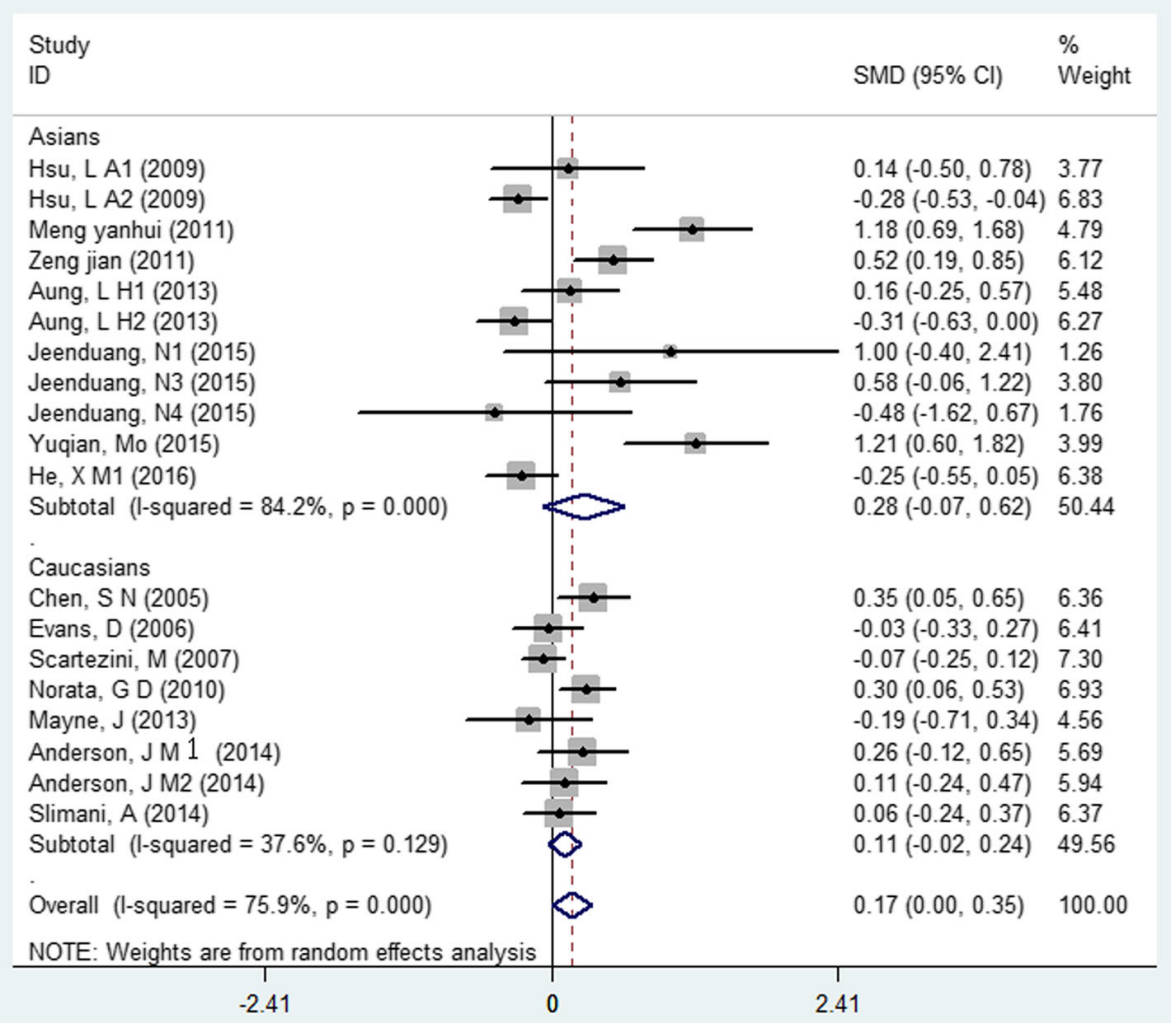
Forest plot of the association between PCSK9 rs505151 polymorphism and serum low-density cholesterol level

As for *PCSK9* rs505151, pooled results under dominant genetic model (AG + GG VS AA) indicated that G allele carriers had higher TG levels in Asians (SMD: 0.14, 95% CI: 0.02 to 0.26, *P* = 0.021, I^2^ = 0) and higher LDL-C levels (SMD: 0.17, 95% CI: 0.00 to 0.35, *P* = 0.046, I^2^ = 75.9%. Fig. 2), the grade of evidence were identified as “moderate” and “weak”, respectively. No Statistical association was found between *PCSK9* rs505151 variant and TC, HDL-C, diastolic blood pressure (DBP) and systolic blood pressure (SBP) Table 3.

**Fig. 3:**
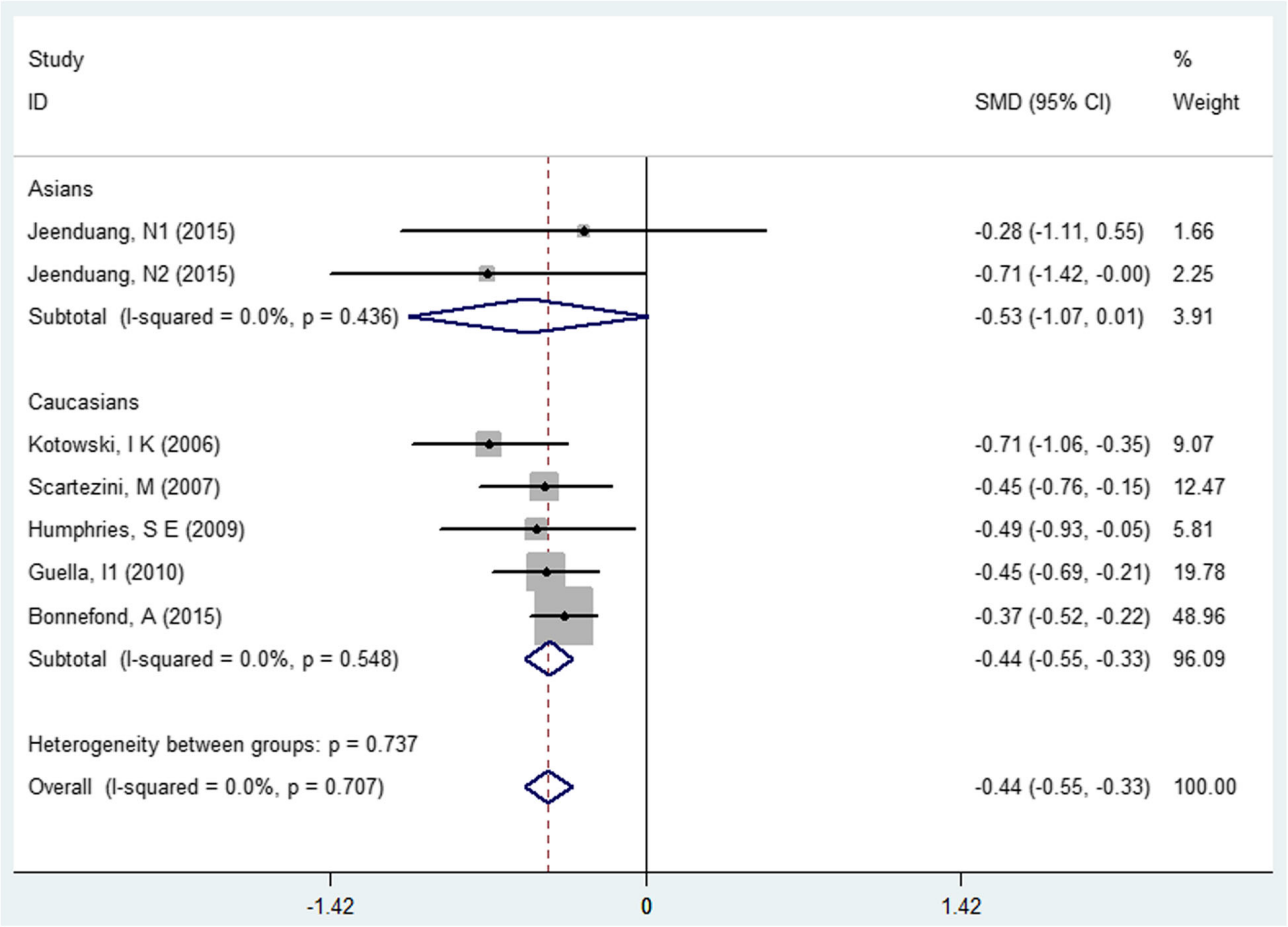
Forest plot of the association between PCSK9 rs11591147 polymorphism and serum low-density cholesterol level

Findings showed that T allele in rs11591147 was significantly associated with lower TC level and LDL-C level in Caucasians (TC, SMD: -0.45, 95% CI: -0.57 to −0.32, *P* = 0.000, I^2^ = 0; LDL-C, SMD: -0.44, 95% CI: -0.55 to −0.33, *P* = 0.000, I^2^ = 0, Fig. 3), the credibility of the both pooled findings were identified as “moderate”. We did not find any statistical significant association of the PCSK9 rs11591147 polymorphism with TG, HDL-C, DBP and SBP Table 3.

#### Associations of the *PCSK9* rs505151 and rs11591147 polymorphisms with cardiovascular risk

**Fig. 4:**
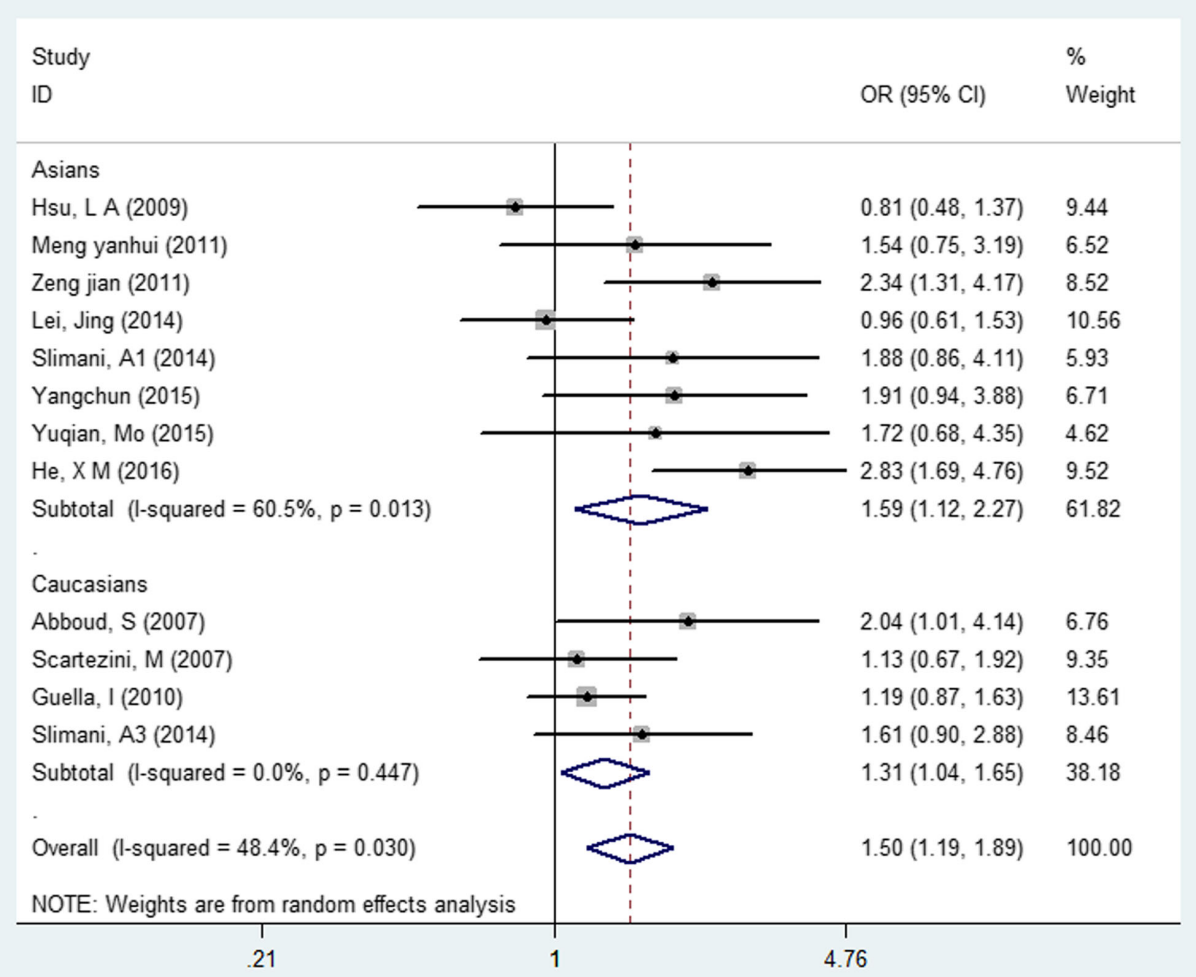
Forest plot of the association between PCSK9 rs505151 polymorphism and cardiovascular risk

**Fig. 5:**
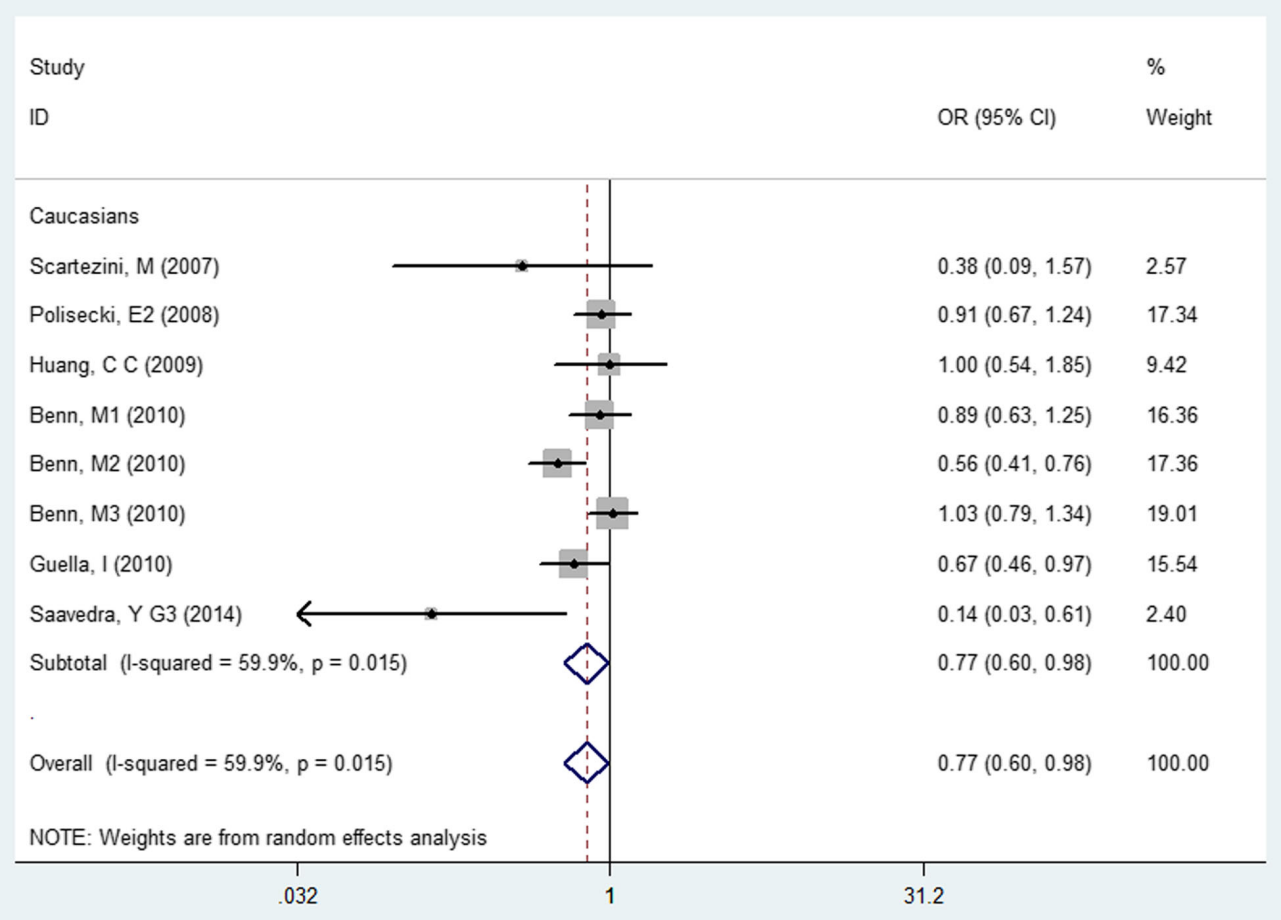
Forest plot of the association between PCSK9 rs11591147 polymorphism and cardiovascular risk

As shown in Fig. 4 and Table 4, the dominant model of G allele in *PCSK9* rs505151 was significantly increased cardiovascular risk (OR:1.50, 95% CI: 1.19 to 1.89, *P* = 0.0006, I^2^ = 48%), the evidence was identified as “moderate”; subgroup meta-analysis, which stratified by ethnicity presented consistent results (Asians, OR:1.59, 95% CI: 1.12 to 2.27, *P* = 0.009, *I* ^*2*^ = 60; Caucasians, OR:1.31, 95% CI: 1.04 to 1.65, *P* = 0.02, I^2^ = 0). Conversely, the T allele of rs11591147 related to decreased cardiovascular risk in Caucasians (OR: 0.77, 95% CI: 0.60 to 0.98, *P* = 0.031, I^2^ = 59.9, Fig. 5), the evidence was graded as “weak” because of large between-study various (I^2^ = 63.2%).

#### Heterogeneity and publication bias

Some heterogeneity was observed in the meta-analysis. Among these statistical Significant findings, the associations of *PCSK9* rs505151 polymorphism with increased serum TG concentration and increased cardiovascular risk, the associations between *PCSK9* rs11591147 polymorphism and decreased cardiovascular risk were based on heterogeneous data, which lowered the credibility of pooled evidence. Begg’s and Egger’s tests were applied to detect the potential publication bias, data showed there was no potential publication bias in all the comparisons except one meta-analysis about the association of rs11591147 polymorphism and plasma HDL-C level (*P* = 0.026 for Egger’s tests). More details were reported in Table 4.

## Discussion

In the current study, we performed the most comprehensive meta-analysis of the associations of the rs505151 and rs11591147 functional mutations of *PCSK9* with serum lipids level and cardiovascular risk that has been conducted to date. Synthetic results clearly showed an association between the G allele of *PCSK9* rs505151 and increased serum LDL-C levels; this is the first time that the relationship between *PCSK9* rs505151 variants and increased TG concentrations has been demonstrated. Furthermore, this single nucleotide polymorphism (SNP) was also shown to be related to an increased incidence of cardiovascular events. Conversely, the T allele of the *PCSK9* rs11591147 variation was found to be associated with reduced serum TC and LDL-C levels and strongly related to a reduction in cardiovascular risk among the general Caucasian population. According to the Venice criteria [15], the credibility of all the nominally statistically significant associations was in the range “moderate” to “weak”, predominantly because of the observed between-study heterogeneity.

Previous meta-analyses have shown the association of *PCSK9* rs505151 variants with increased serum TC and LDL-C levels, as well as increased cardiovascular risk [17–20]; however, the present meta-analysis revealed that this SNP was closely related to higher LDL-C and TG levels, but not with higher TC levels. The role of PCSK9 in cholesterol regulation is well-recognized; however, this study is the first to demonstrate the association between *PCSK9* rs505151 variants and serum TG levels, although it remains to be determined whether this relationship is causal or a concomitant phenomenon. As for *PCSK9* rs11591147, a meta-analysis has been reported that the *PCSK9* 46 L allele was associated with reductions in LDL-C and ischemic heart disease via pooled three independent studies in 2010 [21]. Given the discrepancies in the results reported in recent years, this meta-analysis was performed to explore the true associations of *PCSK9* rs11591147 variants with lipid levels and cardiovascular risk; the consistency of the results of this meta-analysis further confirmed the robust relationship.

Since it was first reported in 2003, PCSK9 has attracted a lot of attention regarding its key role in lipid metabolism. PCSK9 enhances LDLR lysosomal degradation, resulting in reduced LDL-C clearance, thereby leading elevated serum LDL-C levels [22, 23]. Functional mutations of PCSK9 could have a real impact on serum lipids level and cardiovascular risk. The rs505151 and rs11591147 variants of *PCSK9* are classified as GOF- and LOF-mutations, respectively. Notably, this study further confirmed the association of rs505151 with increased LDL-C levels and cardiovascular risk, while there was a strong association of the rs11591147 polymorphism with reduced LDL-C levels and cardiovascular risk. The *PCSK9* gene is highly polymorphic, and functional variants affect the activity of the PCSK9 protein, resulting in lipid metabolism disturbances. Despite the less marked effect of a single SNP on the pathophysiological processing, adding genetic information to lipid management and cardiovascular risk prediction may be potentially useful in clinical practice. Pharmacogenetic studies have shown that GOF and LOF variants of PCSK9 are associated with worse and better responses to statin therapy, respectively [24, 25]. The most likely reason for this is that these functional variants of PCSK9 cause disturbances in cholesterol metabolism and lead to higher or lower cholesterol concentrations, respectively. Despite the current lack of genetic tests to guide statin therapy, the findings of this study could provide useful information for determining the optimal therapy. For instance, standard statin treatment fails to achieve cholesterol targets in some patients. In such cases, administration of a PCSK9 inhibitor would preferable to increasing the statin dose, regardless of knowledge of the patient’s genotype. Furthermore, combining identified variants, such as *PCSK9* rs505151, into risk prediction models, may show some improvement in cardiovascular risk prediction for primary prevention. Unlike the traditional factors, genetic variants, if they can be identified, may be strong predictors with lifelong value in preventive management.

Some limitations of this meta-analysis should be noted. First, heterogeneity in the data may reduce the credibility of the pooled evidence. The main factors responsible for this heterogeneity were study design (cohort or case-control) and genotyping method (Taq Man or PCR-RFLP), which is a common problem in genetic meta-analyses. Therefore, the adoption of strict standards will be encouraged in performing clinical studies. Second, only one genetic model (dominant model) was applied in our analysis, as explained in the Materials and methods section. However, the use of different models would have increased the number of meta-analyses, with consequent inflation of the type I error [26]. Third, the size of the Asian population included in this meta-analysis of *PCSK9* rs11591147 was small, and pooled results revealed heterogeneity in the Asian group, but not in the Caucasian group, indicating that more high-quality clinical studies in Asian populations are required. Fourth, most original studies included in the present meta-analysis used the combined cardiovascular event to estimate the cardiovascular risk, thus taking difficult to identify the risk of specific cardiovascular event. In the final, though we have provided evidence support the association between the two SNPs of PCSK9 (rs505151 and rs11591147) with lipid traits and cardiovascular events, we compromised estimates of heritability based on the current data. Accurate estimates of heritability will require more extensive examination of each identified SNP, especially in a scenario where variants are more likely to be causal for traits and disease.

## Conclusions

This study provides evidence that the variant *PCSK9* rs505151 allele confers increased TG and LDL-C levels on the carrier, as well as increased cardiovascular risk. Conversely, the variant *rs11591147* allele protects against CVD susceptibility and is associated with lower TC and LDL-C levels. These findings could provide useful information for researchers interested in PCSK9 and cardiovascular risk prediction not only in the design of future studies, but also improved clinical and public health. However, further investigations are required to identify the biological function of the two *PCSK9* SNPs and to distinguish direct or indirect influences of the variant alleles on cardiovascular risk.

## Abbreviations

ADH: Autosomal dominant form of hypercholesterolemia
CAD: Coronary artery disease
CHD: Coronary heart disease
CI: Confidence interval
CVD: Cardiovascular disease
DBP: Diastolic blood pressure
GOF: Gain-of-function
HDL-C: High density lipoprotein cholesterol
HWE: Hardy-Weinberg equilibrium
IS: ischemic stroke
LDL-C: Low-density lipoprotein cholesterol
LDLR: Low-density lipoprotein-receptor
LOF: Loss-of-function
MAF: Minor allelic frequency
MI: and Myocardial infarction
OR: odds ratio
PCSK9: Proprotein convertase subtilisin/kexin type 9
SBP: Systolic blood pressure
SMD: Standardized mean difference
TC: Total cholesterol
TG: Triglyceride
